# How post-translational modifications in pathogenic fungi inform pathogenesis and immune responses

**DOI:** 10.1371/journal.ppat.1014162

**Published:** 2026-05-08

**Authors:** Satya Ranjan Sahu, Charles A. Specht, Stuart M. Levitz

**Affiliations:** Department of Medicine, University of Massachusetts Chan Medical School, Worcester, Massachusetts, United States of America; University of Maryland, Baltimore, UNITED STATES OF AMERICA

Post-translational modifications (PTMs) are conserved, tightly regulated processes that introduce chemical groups or remove portions of the protein, ensuring structural and functional competence. PTMs regulate protein folding, stability, localization, and activity, particularly of secreted proteins. In pathogenic fungi, these modifications influence signaling, membrane function, cell wall architecture, and gene expression, thereby shaping virulence and host interactions. More than 200 PTMs have been described in eukaryotes, many of which are shared between fungi and humans [1]. This mini review summarizes major PTMs in pathogenic fungi, with an emphasis on how these PTMs, particularly glycosylation, inform immune responses.

## 1. Overview of major PTMs in fungi

**Fig 1:**
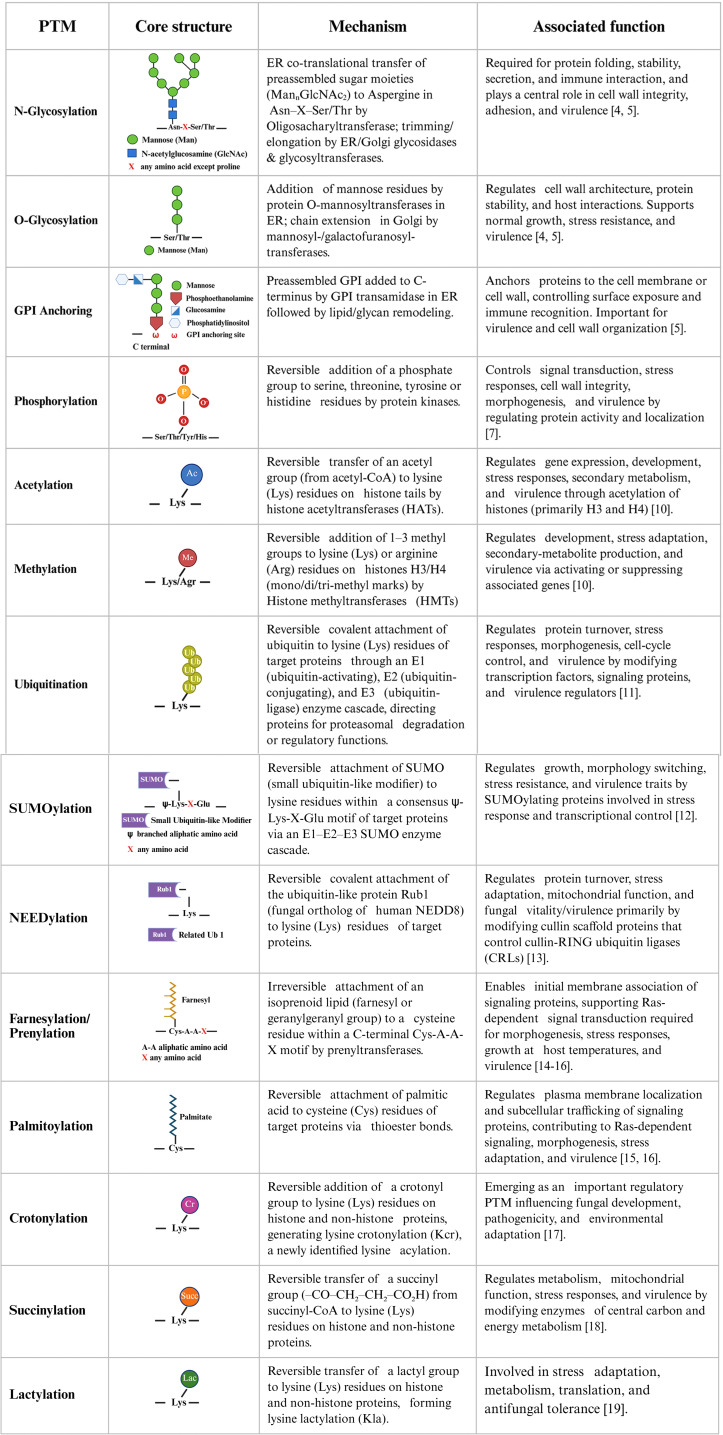
A) and B) List of post-translational modifications in fungi with their core structures, mechanisms, and associated functions. Created in BioRender. Sahu, S. (2026) https://BioRender.com/04u7e1x [20].

The ability of pathogenic fungi to survive, adapt, and cause disease or interact with the host relies heavily on PTMs. This is particularly relevant for cell wall architecture and integrity [2]. Various PTMs such as glycosylation, phosphorylation, acetylation, methylation, ubiquitin-related modifications, and lipid modifications have been reported in fungi [3]. While glycosylation has been well studied, the involvement of other PTMs in fungal pathogenicity and host–pathogen interactions remain poorly understood. An outline of fungal PTMs, with core motifs, mechanism, and associated functions, is provided in Fig 1A and 1B, while major modifications are discussed in detail below.

Glycosylation involves the addition of N-linked and O-linked glycans, as well as glycosylphosphatidylinositol (GPI) anchors, mediated by distinct glycosyltransferases. In N-glycosylation, a preassembled oligosaccharide is transferred to an asparagine (Asn) within the consensus Asn-X-Ser/Thr motif (Ser, Serine; Thr, Threonine; X, any amino acid except Proline) on nascent proteins. Whereas O-glycosylation involves the addition of mannose residues to Ser or Thr; there is no motif that determines which Ser or Thr is mannosylated. GPI anchor synthesis and its attachment to the C-terminal region of a protein, known as the omega (ω) site, are conserved processes across eukaryotes. The key distinction between fungal and mammalian glycosylation lies in glycan composition: protein-linked glycans in fungi are enriched in mannose and terminally mannosylated, whereas mammalian N-glycans rarely add mannose to the oligosaccharide core and are extensively processed into complex or hybrid forms containing galactose, fucose, or sialic acid. In contrast, mammalian O-glycans are very heterogeneous with mannose, fucose, glucose, xylose, N-acetylglucosamine, or N-acetylgalactosamine transferred to Ser or Thr followed by further additions of other monosaccharides [4]. Differences in sugar utilization, glycan length, and branching are frequently seen when comparing protein glycosylation from different fungal species. Intraspecies and morphotype-specific variations in glycan structure are also seen. Disruption of glycosylation compromises cell wall architecture, reducing fungal fitness and virulence. For example, the α-1,6-mannosyltransferase Och1 (outer-chain initiation protein 1) initiates outer-chain mannosylation of N-glycans by adding the first mannose required for chain extension. In the absence of Och1, N-glycans remain truncated rather than fully elongated. Fully N-glycosylated, mannose-rich glycans on cell wall proteins help form a thick outer coat that maintains cell wall rigidity and limits access of host lytic enzymes. Therefore, *och1Δ* mutants show a weakened cell wall, reduced fungal fitness, increased susceptibility to host attack, and ultimately attenuated virulence in major pathogenic fungi like *Candida albicans*, *Aspergillus fumigatus*, and *Cryptococcus neoformans* [5,6].

Phosphorylation is another major PTM characterized by the addition of a phosphate group to serine, threonine, or tyrosine residues by kinases. It acts as a rapid, reversible signaling switch, enabling fungi to sense environmental changes and regulate morphogenesis, stress tolerance, and virulence factors like capsules and pigments [7]. Deletion of Tpk2 (cAMP-dependent protein kinase catalytic subunit 2) in *C. albicans* impairs filamentation and virulence, whereas loss of Pka1 (Protein kinase A catalytic subunit 1) abolishes capsule and melanin production in *C. neoformans* [7–9].

Epigenetic PTMs, particularly histone acetylation and histone methylation, regulate chromatin accessibility and transcriptional programs linked to stress adaptation and virulence. Histone acetylation, the addition of acetyl groups to lysine residues, relaxes chromatin and generally promotes transcription, whereas histone methylation, the addition of methyl groups to lysine or arginine residues, can activate or repress gene expression depending on the modified site [10]. These mechanisms enable rapid transcriptional reprogramming under host-derived stresses such as oxidative damage and nutrient limitation. Important histone acetyltransferases like Gcn5 (General control non-derepressible protein 5) regulate morphogenesis and stress tolerance, while the conserved H3K4 methyltransferase Set1 (SET domain–containing protein 1) governs yeast-to-hypha transitions and stress responses [10].

Ubiquitin-related PTMs, including ubiquitination, SUMOylation, and NEDDylation, shape fungal pathogenicity by regulating protein stability, localization, and signaling dynamics. These modifications involve the covalent attachment of ubiquitin or ubiquitin-like proteins (such as SUMO and NEDD8/Rub1) to the protein. Ubiquitination enables rapid proteome remodeling during stress and infection [11], SUMOylation regulates nuclear processes and transcription [12], and NEDDylation modulates cullin-based ubiquitin ligase activity [13].

Lipid modifications such as prenylation and palmitoylation control membrane association of key signaling proteins and regulators of chitin synthesis [14]. Sequential prenylation and palmitoylation of Ras1 (Ras-like small GTPase 1) are required for membrane localization and activity, and loss of these modifications results in thermal sensitivity, abnormal morphogenesis, and avirulence in murine infection models [15,16].

Newly studied acylation PTMs in fungi involve the addition of acyl groups such as crotonyl, succinyl, and lactyl to lysine residues on proteins. Although their roles in fungal–host interactions and virulence remain incompletely understood, current studies indicate that they regulate essential cellular processes. Crotonylation in *C. albicans* is more prevalent than acetylation and succinylation and can be predicted using a fungal-specific language model, Fungi-Kcr [17]. Succinylation has reported functional roles in *C. albicans* and *S. cerevisiae* and mediates aflatoxin biosynthesis in *A. flavus*. Lactylation is prominent in *C. albicans* ribosomal proteins, central carbon metabolism enzymes, and core histones (H2A–H4) [18,19].

## 2. How cell wall-associated PTMs impact host immune recognition

**Fig 2:**
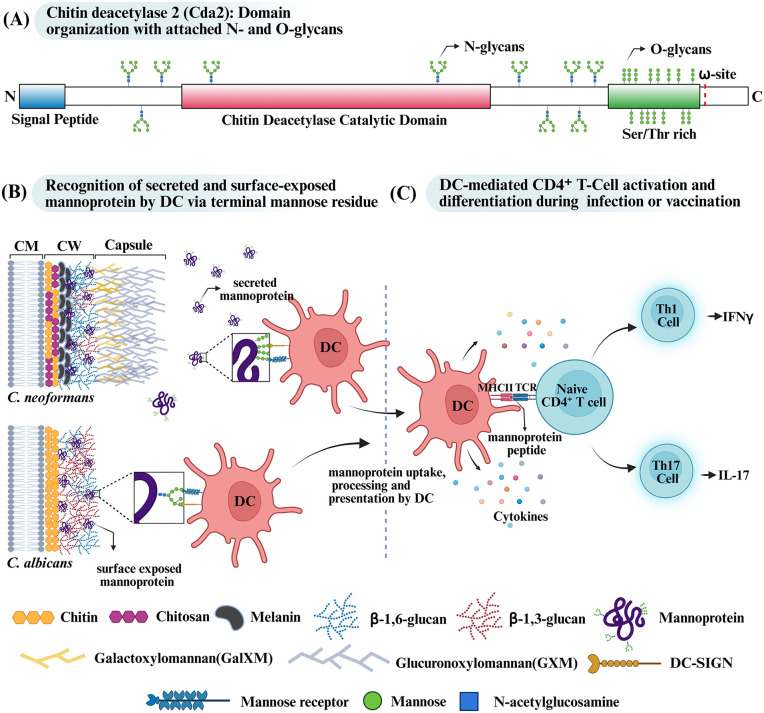
(A) Schematic representation of *C. neoformans* chitin deacetylase 2 (Cda2), showing an N-terminal signal peptide, a central catalytic deacetylase domain, and a C-terminal serine/threonine (ST)-rich region containing an ω-site for glycosylphosphatidylinositol (GPI) attachment, with N-linked and O-linked glycans distributed across the protein. **(B)** Secreted mannoproteins from *C. neoformans* and surface-exposed mannoproteins from *C. albicans* are recognized by dendritic cells (DCs) via the mannose receptor (CD206, MRC1) and DC-SIGN (CD209), which bind terminal mannose residues on N- and O-linked glycans and mediate antigen uptake. **(C)** Following antigen uptake and processing, DCs present mannoprotein-derived peptides on MHC class II molecules to naive CD4⁺  T cells, leading to T-cell activation and differentiation into Th1 and Th17 subsets, characterized by the production of IFN-γ and IL-17, respectively. Created in BioRender. Sahu, S. (2026) https://BioRender.com/04u7e1x [20]. CM; Cell membrane, CW; Cell Wall, DC-SIGN; Dendritic Cell-Specific Intercellular adhesion molecule-3-Grabbing Non-integrin, MHCII; Major Histocompatibility Complex class II, TCR; T-cell receptor IL-17; Interleukin and IFNγ; Interferon-gamma.

The fungal cell wall functions as both a physical barrier and a critical immunological interface. As the first point of host contact, PTMs that alter cell wall composition or surface exposure strongly influence pattern-recognition receptor (PRR) engagement, cytokine production, and downstream immune responses. Glycosylation of proteins with highly branched N-mannans generates a dense outer glycan coat that masks immunogenic β-glucans from Dectin-1 and other host β-glucan receptors and protects the cell wall from host proteases and lytic enzymes [21]. At the same time, mannans act as pathogen-associated molecular patterns (PAMPs) and are recognized by mannose-specific C-type lectin receptors. An illustrative example of cell wall PTMs impacting immune recognition is *C. neoformans* Cda2 (chitin deacetylase 2/MP98). This mannoprotein is extensively N- and O-glycosylated and has a GPI anchor, enabling plasma membrane as well as cell wall localization. Fig 2A offers a schematic overview of Cda2's structural features, including glycosylation sites and its GPI anchoring site, underscoring how these modifications can direct immune engagement [22]. The ability of Cda2 to act as an immunodominant antigen capable of stimulating T cell–mediated responses depends on recognition of O-linked and N-linked mannans by the mannose receptors CD206 (MRC1) and CD209 (DC-SIGN) on antigen-presenting cells [23,24]. As *C. neoformans* possesses a polysaccharide capsule, most cell wall-associated mannoproteins remain beneath the capsule and are less accessible for direct immune recognition. Consequently, host immune interactions are primarily mediated by secreted mannoproteins. In contrast, other pathogenic fungi such as *C. albicans* have exposed cell wall-associated mannoproteins on their surface, where they can directly serve as ligands for mannose receptors. Both secreted and cell wall-associated mannoproteins can be recognized and taken up by host antigen-presenting cells via receptor-mediated mechanisms. This uptake leads to processing and presentation of mannoproteins as antigens to CD4⁺ T cells, initiating downstream immune processes such as activation and differentiation of CD4⁺ T cell subsets into Th1 and Th17 cells. Fig 2B and 2C illustrate these immunological events from mannoprotein recognition, through antigen processing and presentation, to the activation and differentiation of CD4⁺ T-cell subsets, resulting in cytokine secretion. Disruption of mannosylation pathways across fungal species further underscores the immunological importance of glycan PTMs. In *C. albicans*, *och1ΔΔ* mutants exhibit reduced TNF-α and IL-6 production by peripheral blood mononuclear cells and impaired trained immunity, while *mnt1ΔΔ* (mannosyltransferase-1) mutants show defective epithelial adherence, increased susceptibility to macrophage killing, and loss of virulence [5,25]. In *C. neoformans*, deletion of *PMT1* or *PMT4* (*protein O-mannosyltransferase-1/4*) increases susceptibility to macrophage-mediated killing, and the *pmt1Δ pmt4Δ* double mutant is synthetically lethal, highlighting the essential role of O-mannosylation in fungal survival and immune evasion [5].

## 3. Secretion and shedding of fungal mannoproteins—pathways and consequences for immune activation

Most fungal cell wall mannoproteins are synthesized via the classical ER–Golgi secretory pathway and are directed to the cell membrane or cell wall by an N-terminal signal peptide and a C-terminal GPI anchor. At the cell wall, many mannoproteins are covalently linked to β-1,6-glucans, forming the outer fibrillar layer that contributes to structural integrity and antigen masking [26]. However, proteomic and biochemical studies have shown that a substantial fraction of these proteins is released into the extracellular environment through cell wall remodeling, proteolytic cleavage, or extracellular vesicle (EV)–mediated transport [27,28]. As a result, mannoproteins exist in functionally distinct pools: cell wall–bound, soluble or EV-associated forms, each contributing differently to fungal biology and host immune responses.

Well-characterized mannoproteins include Als3, Hwp1, and Mp65 in *C. albicans* [29]; Cda family proteins and MP88 in *C. neoforman*s [22]; and Afmp1p/Afmp2p in *A. fumigatus* [5]. In their wall-associated state, these proteins support cell wall architecture and perform protein-specific functions such as enzymatic activity and facilitating adhesion or invasion. In contrast, released mannoproteins act as potent immunogens that stimulate cytokine production and immune responses in the host. In *C. neoformans*, Cda family proteins catalyze the conversion of chitin to chitosan, a process essential for cell wall integrity, capsule organization, and virulence. Deletion of multiple *CDA* genes results in rapid fungal clearance in murine models, revealing the importance of chitosan and Cda’s enzymatic activity [30,31]. The immunological properties of secreted Cdas are strongly shaped by glycosylation. Secreted Cda2 and Cda3 purified from wild-type strains and *alg3Δ* (α-1,3-mannosyltransferase 3) mutants, which produce truncated N-glycans, showed no major differences in epithelial adhesion or macrophage uptake, but *alg3Δ*-derived proteins induced significantly lower TNF-α production [32]. Conversely, mutation of all N-glycosylation sites in Cda3 enhanced epithelial adhesion, macrophage uptake, and cytokine induction, while removal of O-glycans markedly reduced CD4⁺ T-cell activation [32]. Thus, despite both being mannose-rich, N- and O-glycans elicit different responses by the host immune system, with O-glycosylation appearing to play a dominant role in immune activation, although precise contributions remain to be fully defined.

## 4. Implications of PTMs for antifungal therapies and glycoprotein-based vaccine strategies

Targeting PTMs offers a promising strategy and opens new dimensions for exploring novel antifungals. Since many PTM pathways are shared by both fungi and humans, the best therapeutic approach is to target PTM enzymes that are unique to fungi or sufficiently different from their human counterparts, thereby reducing host toxicity. The Gwt1 (a GPI anchor biosynthesis enzyme) inhibitor fosmanogepix is in phase III clinical trials for the treatment of invasive fungal infections [5]. Pradimicins, a class of mannose-binding broad-spectrum antifungals, reached preclinical and early phase I investigations but never progressed to phase II trials. Wortmannin exhibits antifungal activity with a mechanism of action that appears to involve inhibition of two PTMs, phosphoinositide 3-kinase (PI3K) and Kre2/Mnt1 [5]. These examples collectively highlight how PTM-targeting drugs can be broadly relevant across fungal pathogens.

Vaccine development has traditionally focused on antigens required for pathogen survival or virulence. Cryptococcal mannoproteins are essential virulence-associated and highly immunogenic cell wall components [33]. Mannoprotein-driven T-cell activation has therefore been exploited in vaccine studies to determine whether the induced immune responses translate into antigen-specific T cells protective immunity. Adjuvanted vaccines containing mannoprotein-rich fractions from *C. neoformans* were shown to confer protection in mice. Chemical deglycosylation of O-linkages markedly reduced protective efficacy, providing evidence for a critical role for glycosylation, presumably by promoting uptake of antigens by mannose receptors on antigen-presenting cells [34]. A mild alkaline extract of *C. neoformans* cells has been used as a vaccine antigen when combined with glucan-particles (GP), inducing robust Th1/Th17-biased CD4⁺ T-cell responses in the lungs of vaccinated and infected mice [35]. Mass spectrometric analysis of these alkaline extracts revealed the presence of multiple mannoproteins, including members of the Cda family and MP88. Key mannoproteins were subsequently expressed recombinantly in *E. coli* and evaluated as subunit vaccines. Notably, Cda1 and Cda2 delivered in GP-based vaccines conferred strong protection across multiple mouse models, inducing CD4⁺ T-cell responses [36]. These results demonstrate that the protein backbone alone can induce protective immunity, without native glycans, if formulated with an adjuvant that promotes strong CD4⁺ T-cell responses. Consistent with this, protection was lost in mice that lacked CD4⁺ T-cells but retained in CD8⁺ T-cell–deficient mice [37].

While *C. neoformans* mannoproteins are masked by the polysaccharide capsule, other medically important fungi such as *C. albicans* expose mannans directly on their cell surface, making them attractive vaccine antigens. *C. albicans*’s surface mannans can induce antibodies that inhibit adhesion, promote opsonophagocytosis, neutralize toxins, and directly counter fungal growth [38].

A well studied vaccine target in *C. albicans* is the agglutinin-like sequence 3 protein (Als3p), a highly glycosylated, hypha-associated adhesin and invasin. The NDV-3A vaccine, which contains the recombinant N-terminal domain of Als3p (rAls3p-N), elicits anti-Als3p antibody and Th1/Th17-biased CD4⁺ T-cell responses, and protects in mouse models of candidiasis [39]. In a Phase 2 trial of women with recurrent vulvovaginal candidiasis, vaccination with NDV-3A reduced the frequency of symptomatic episodes in the subset of subjects aged under 40 years [39,40]. A fully synthetic glycopeptide vaccine against disseminated candidiasis combined β-1,2-linked mannotriose [β-(Man)₃], a mimic of *C. albicans* cell wall mannans with weak intrinsic immunogenicity, with peptide epitopes derived from cell wall proteins including fructose-bisphosphate aldolase (Fba), methyltetrahydropteroyltriglutamate (Met6), and hyphal wall protein-1(Hwp1). Among these constructs, the β-(Man)₃–fructose-bisphosphate aldolase conjugate induced strong antibody-mediated protection, as demonstrated by passive serum transfer to naive mice, with protection dependent on immune responses to both the carbohydrate and peptide epitopes [41].

Beyond *Candida* and *Cryptococcus*, PTM-related targets have also been explored in Mucorales fungi. The CotH (spore coat protein homolog), a GPI-anchored surface protein, facilitates angioinvasion and dissemination in Mucorales by interacting with the host receptor GRP78 (glucose-regulated protein 78), a process intensified in diabetic ketoacidosis (DKA). Anti-CotH antibodies can disrupt this interaction, reduce endothelial invasion, and significantly improve survival in DKA mouse models of mucormycosis [42]. Building on these findings, a humanized IgG1 monoclonal antibody was developed, which improved antigen binding, reduced vascular injury, and enhanced fungal clearance [43].

## 5. Unanswered questions and future directions for the field

Despite advances in fungal PTM research, major knowledge gaps remain. Most PTMs, apart from glycosylation, are poorly explored in the context of host–pathogen interactions, and even this well studied modification remains incompletely understood with respect to its regulation under host-induced stress and its impact on antifungal efficacy [44]. It is also unclear if cell wall mannoproteins undergo additional PTMs beyond glycosylation, GPI anchor attachment and proteolysis. Moreover, it remains unknown whether fungi modulate N- and O-glycan density on mannoproteins in response to distinct host niches such as the lungs, brain, or gut. While mannosylation is known to enhance antigen uptake via receptor-mediated endocytosis, the effect PTMs have on antigen processing and presentation is largely unstudied despite their important implications for immune responses and vaccine development. Importantly, studies of fungal proteins using recombinant proteins made in *E. coli* will miss effects PTMs have on the structure, function, and antigenicity of the native proteins.

Given the conservation of N-glycosylation and GPI anchor attachment motifs, programs for these additions can be searched for using NetNGlyc, big-PI Fungal Predictor, respectively. However, PTM prediction programs such as NetOGlyc (O-glycans) are trained on mammalian datasets, limiting their accuracy for fungal proteins [45]. Some in silico tools for predicting PTMs in yeast, such as NetPhosYeast (phosphorylation), are available. A caveat is prediction tools trained on one fungus, such as *S. cerevisiae*, may not be accurate with other fungal species. Moreover, in silico predictions still require experimental validation, typically with mass spectrometry. Emerging multi-omics, bioinformatics, and artificial intelligence/machine learning collectively hold great promise for addressing key questions in understanding fungal PTMs and identifying novel antifungal candidates targeting PTMs [46,47]. Financial support from government and private funders is essential to maintaining and expanding these key community resources. With the growing global burden of fungal infections and rising drug resistance, integrating advanced tools to systematically study fungal PTMs offers a promising path to close critical knowledge gaps and drive breakthroughs in diagnostics, therapeutics, and vaccine design.
